# Skeleton-based rehabilitation movement quality classification: a leakage-controlled benchmark on IntelliRehabDS

**DOI:** 10.3389/fmed.2026.1878438

**Published:** 2026-07-15

**Authors:** Zhen Zhu, Yuqiong Xiang, Xianzhu Tian

**Affiliations:** 1 Department of Orthopedics, Jiujiang Hospital of Traditional Chinese Medicine, Jiujiang, Jiangxi, China; 2 School of Rehabilitation Medicine, Gannan Medical University, Ganzhou, Jiangxi, China

**Keywords:** class imbalance, data leakage, IntelliRehabDS, machine learning benchmarking, rehabilitation movement classification, skeleton-based action quality assessment, subject-independent evaluation

## Abstract

**Introduction:**

Automated, objective assessment of rehabilitation movement quality from three-dimensional skeleton recordings can support scalable and low-cost physiotherapy monitoring, but reported performance is often affected by subject leakage, inconsistent class-imbalance handling and non-comparable evaluation protocols.

**Methods:**

This study establishes a leakage-controlled, subject-independent benchmark on the publicly available IntelliRehabDS corpus. After removing 12 ambiguous-label files and 2 sequences shorter than 16 frames, 2,575 sequences from 29 adult and adolescent participants were retained, comprising 2,047 correct and 528 incorrect executions across nine rehabilitation gestures. Sequences were root-centred, torso-scaled, smoothed with a Savitzky–Golay filter, resampled to 64 frames and represented through kinematic features or skeleton tensors. Five classical feature-based learners and the topology-aware ST-SkelNet sequence model were evaluated through nested subject-wise cross-validation. Scaling and SMOTE were fitted only within the relevant training partitions for the classical models, whereas ST-SkelNet used class-weighted binary cross-entropy.

**Results:**

Performance was reported using fold-averaged metrics, pooled out-of-fold confusion matrices and ROC-AUC analysis. No single model dominated: the multilayer perceptron achieved the highest accuracy (0.869), the random forest the highest ROC-AUC (0.861), and the support vector machine the highest incorrect-class recall (0.629). ST-SkelNet achieved 0.846 accuracy, 0.855 ROC-AUC and 0.612 incorrect-class recall, but did not consistently exceed the strongest classical baselines. Head-motion and bilateral-asymmetry features showed descriptive separation between correct and incorrect executions in this adult/adolescent cohort.

**Discussion:**

The contribution is a reproducible methodological benchmark rather than a clinical system or a state-of-the-art architecture. IntelliRehabDS is treated here as a surrogate adult/adolescent dataset for methodological validation and benchmarking, not as infant data. Transfer to paediatric populations, including congenital muscular torticollis, requires independently collected age-specific data and prospective clinical validation.

## 1 Introduction

Automatic assessment of the quality of rehabilitation movements from three-dimensional skeleton recordings has become an active research area, driven by the availability of low-cost depth sensors that stream full-body joint coordinates at 30 frames per second. Such skeleton streams are privacy-preserving, compact and largely invariant to appearance and illumination, which makes them attractive for objective, scalable monitoring of physiotherapy both in the clinic and at home. The central computational task is to distinguish correctly executed therapeutic movements from incorrect ones, or to grade the quality of execution, using only the geometry and temporal dynamics of the moving skeleton.

Despite rapid methodological progress, three problems repeatedly undermine the comparability and credibility of reported results. First, many evaluation protocols allow sequences from the same person to appear in both the training and test partitions; because skeleton geometry is strongly subject-specific, this leakage produces optimistic estimates that do not reflect performance on unseen individuals. Second, correctness labels in rehabilitation corpora are typically imbalanced, with incorrect executions in the minority, yet imbalance handling is often applied before rather than inside the cross-validation folds, which itself leaks information. Third, comparisons across studies mix different datasets, tasks and metrics, so headline numbers are rarely commensurable. A rigorous benchmark therefore requires strictly subject-disjoint evaluation, leakage-controlled preprocessing and imbalance handling, and a single shared protocol under which classical and sequence-based models can be compared on equal terms.

Within the broader rehabilitation literature, automatic movement quality assessment based on three-dimensional skeleton recordings has matured considerably over the last few years (1). Depth sensors of the Kinect family deliver a streamable 30-frames-per-second skeleton at low cost, and graph convolutional networks, transformers and recurrent architectures have been used to grade or classify exercise execution on benchmarks such as KIMORE and UI-PRMD (2). A 2025 systematic review of depth-camera physiotherapy assessment confirms that machine learning can reproduce expert scoring with clinically meaningful agreement when feature engineering is tightly aligned with the target movement (3).

A shared, leakage-controlled correctness benchmark for these methods is, however, still missing. Most existing rehabilitation datasets focus on the elderly, on stroke survivors or on adult low-back pain protocols (4), and studies built on the IntelliRehabDS corpus have largely targeted gesture recognition rather than the binary correctness label, so the movement-quality dimension remains under-explored (5). The IntelliRehabDS dataset is well suited to fill this gap: it comprises 29 distinct adult and adolescent subjects executing nine clinically meaningful upper-limb gestures, each annotated as correctly or incorrectly executed by two independent annotators, and recorded across sitting, standing and wheelchair postures (5).

The aim of the present study is to establish a reproducible, subject-independent benchmark for skeleton-based rehabilitation movement-quality classification on IntelliRehabDS. IntelliRehabDS is used here as a surrogate adult/adolescent benchmark dataset on which the methodology is developed and stress-tested; it contains no infant recordings. The study does not claim a new clinical system for congenital muscular torticollis or state-of-the-art architectural performance. Its methodological contribution comprises: (i) a subject-disjoint evaluation protocol; (ii) nested group-wise hyperparameter selection in which the outer test subjects never influence model selection; (iii) leakage-controlled preprocessing and imbalance handling fitted only within training partitions; (iv) a same-protocol comparison of classical feature-based models and a topology-aware sequence model; (v) complete pooled out-of-fold confusion-matrix and ROC-AUC reporting; and (vi) an interpretable analysis of kinematic feature relevance. The results establish the performance and limitations of these modelling strategies within the available adult/adolescent rehabilitation corpus. Evaluation in infantile torticollis is outside the evidential scope of the present experiments and is identified only as a possible direction for independently designed future research (Section 5.1).

Although machine-learning classification of rehabilitation movements from three-dimensional skeleton data is well established, evaluation practice remains inconsistent. Published studies frequently use non-subject-disjoint splits, apply imbalance correction outside the cross-validation loop or compare results obtained from different datasets, target variables and metrics. To our knowledge, no published IntelliRehabDS study has provided a single correctness benchmark that combines identical subject-wise folds, nested model selection, fold-contained preprocessing and complete pooled diagnostic reporting across both classical and sequence-based models. The novelty of the present work is therefore methodological rather than architectural.

As distant clinical motivation only, objective movement-quality assessment of this kind may eventually be relevant to paediatric physiotherapy, including the conservative management of congenital muscular torticollis (6, 7); this connection is purely contextual, no inference about paediatric populations is drawn here, and torticollis appears only as a named target for independently designed future validation (Section 5.1).

We further make explicit that ST-SkelNet is positioned as a sequence-based, topology-aware comparator rather than as a proposed superior architecture: under the present cohort it does not improve accuracy over the multilayer perceptron (0.869 versus 0.846) or AUC over the random forest (0.861 versus 0.855), and no claim of architectural superiority is made anywhere in this manuscript. It is reported as one model within the benchmark so that a sequence model and classical feature-based models can be compared under one identical protocol. Because all experiments are performed on an adult/adolescent rehabilitation cohort, the findings are interpreted only as adult rehabilitation movement-classification evidence; whether the same variables and models are informative in infants requires independent paediatric data and cannot be inferred from the present analysis.

## 2 Related work

This section reviews the technical literature on skeleton-based rehabilitation movement assessment, which is the primary stream relevant to the present benchmark, and then notes paediatric torticollis only briefly as an underexplored future application domain.

On the technical side, recent contributions have refined skeleton-based rehabilitation assessment along several axes. Graph convolutional approaches encode the natural skeletal topology and outperform purely temporal models (2), while spatio-temporal transformers introduce attention pooling that improves generalisation when the number of subjects is limited (8). A 2023 contribution combined dense spatio-temporal graph convolutions with transformer blocks to assess patient rehabilitation quality on KIMORE and reported state-of-the-art rank correlations (9). A frame-topology fusion hierarchical graph model was published in 2025, again highlighting the importance of joint relations rather than isolated coordinates (10). A study in 2024 that employed a transformer model previously used in human action recognition showed that the classification of execution errors offers more useful feedback than continuous quality scores (11). The KINECAL dataset published in Scientific Data expanded the state-of-the-art space towards fall risk assessment, and assessed the validity of low-cost Kinect data for clinical reasoning (12). Finally, a 2025 benchmark of deep architectures for skeleton-based human motion rehabilitation offered a fair comparison across different datasets, and reinforced that deep models (without any skeletal information) begin to plateau in performance at around 50 subjects (13). However, there are still some gaps. The majority of rehab data was gathered on adult or elderly populations and movement repertoire was not related to neck rehab (4). Usually, studies based on IntelliRehabDS corpus are concerned with gesture recognition, and not much on the binary correctness label, while the quality dimension is still under explored (5). Moreover, evaluation protocols often contain sequences from the same person in the train and test sets, which overestimates the results since biometrics of the skeleton are person-specific (14). Finally, paediatric applications such as congenital muscular torticollis remain an essentially unexplored future domain for skeleton-based movement-quality assessment: no public corpus currently provides infant correctness labels, and the head-on-trunk asymmetry and shoulder-girdle range that are clinically relevant in that setting have not been studied with skeleton models on appropriate paediatric data (15). The present work does not enter this domain; it is noted only to indicate where a future, independently collected benchmark would be required.

Table 1 summarises representative recent studies with their datasets, methods, evaluation metrics and reported scores, providing a comparative backdrop for the present contribution.

**Table 1:** Comparison of representative skeleton-based rehabilitation movement assessment studies (2022–2025).

Study	Dataset	Method	Task	Best score
Deb et al. 2022 (2)	UI-PRMD, KIMORE	Graph CNN	Quality scoring	MAD 0.025
Réby et al. 2023 (22)	UI-PRMD, KIMORE	GCN with body landmarks	Quality scoring	MAD 0.022
Mourchid and Slama 2023 (9)	KIMORE	Dense ST-GCN + Transformer	Quality scoring	Spearman 0.92
Marusic et al. 2024 (11)	KERAAL	Skeleton Transformer	Error classification	Acc 0.74
Zhang et al. 2025 (10)	UI-PRMD, KIMORE	Frame topology fusion HGCN	Quality scoring	MAD 0.018
Ismail-Fawaz et al. 2025 (13)	Multiple	Deep architectures benchmark	Quality + classification	Reference benchmark
This work	IntelliRehabDS (*n* = 29)	ST-SkelNet + skeletal GCN	Correctness classification	Acc 0.846, AUC 0.855

MAD, Mean Absolute Deviation; AUC, Area Under the Receiver Operating Characteristic Curve; Acc, Accuracy; GCN, Graph Convolutional Network; ST-GCN, Spatio-Temporal Graph Convolutional Network; HGCN, Hierarchical Graph Convolutional Network. The IntelliRehabDS cohort comprises N, 29 adult/adolescent subjects.

Table 1 is a contextual comparison only. The listed studies differ in dataset (UI-PRMD, KIMORE, KERAAL, multiple corpora), number of subjects (ranging from a few tens to over 50), target task (continuous quality scoring versus correctness or error classification), split strategy (subject-wise versus sequence-wise, with several not reporting subject-independent testing), and reported metric (mean absolute deviation, Spearman correlation, accuracy or AUC). Because the outcomes and evaluation designs differ, the numerical scores in this table cannot be ranked against one another or against the present study. For that reason we draw all quantitative conclusions exclusively from a same-protocol experimental comparison, in which every model is run on identical IntelliRehabDS data, correctness labels, subject-wise folds, preprocessing, tuning framework and evaluation metrics (Section 4.5). The continuous-scoring entries (MAD, Spearman) are included purely to locate the present binary-correctness task within the wider literature, not as a performance baseline.

## 3 Materials and methods

### 3.1 Dataset

The experiments are conducted using a public dataset, IntelliRehabDS (5), that provides a set of 3D skeleton recordings for physical rehabilitation movements. Data were gathered using a Kinect v2 sensor that measures 25 3D positions of the Kinect’s body joints at 30 frames per second. This archive has been released containing 2,589 individual movement files from 29 subjects (15 patients, 14 healthy controls) consistent with the composition reported in the source dataset publication (5). The files are all named by the convention SubjectID, DateID, GestureLabel, RepetitionNumber, CorrectLabel and Position and each file includes the per-frame joint coordinates of one repetition. There are nine clinically relevant gestures: Elbow flexion, Left arm and Right arm, Shoulder flexion, Both arms, Shoulder forward elevation, Left lateral side tap, Right lateral side tap. The correctness label was independently given by two annotators and indicates if the movement was performed in the correct range and pattern. These postures reflect those encountered in routine therapy practice, namely standing, sitting on a chair, sitting in a wheelchair, and sitting or standing within a support frame.

IntelliRehabDS is an adult and adolescent rehabilitation corpus. The recordings used here capture upper-limb and trunk rehabilitation gestures performed by adult and adolescent participants, and the dataset is used only as a benchmark for skeleton-based classification of rehabilitation-movement quality. IntelliRehabDS serves solely as a surrogate methodological benchmark for evaluating the computational protocol; it is not a biological or clinical proxy for infant biomechanics or congenital muscular torticollis. The data contained in this paper are of adults and adolescents, and so conclusions can be drawn regarding the classification of movement for adult/adolescent rehabilitation, if applicable, but not for paediatric rehabilitation – this will be treated only as future work (Section 5.1).

### 3.2 Pre-processing pipeline

The pre-processing pipeline performs five sequential operations and was implemented in Python 3.10 on a Kaggle environment with a graphics-processing-unit-enabled runtime; the same code can be executed on a standard central processing unit because the operations are not deeply parallel. The pipeline first parses every filename and rejects any sample where the parsing fails. It then loads the comma-separated joint matrix and reshapes it into a tensor of shape T × 25 × 3, where T is the number of frames. Sequences with fewer than 16 frames are discarded because they do not contain a complete repetition, and frames where every joint coordinate is numerically zero are discarded because they correspond to tracking failures.

Each retained sequence is normalised: All frames are translated so the SpineBase joint is at the origin, and coordinates are scaled by the average length of the torso (the distance from the SpineBase to Neck joint) across the sequence. This step eliminates the need of the subject height and absolute distance to the sensor. Along the time axis of each joint and each coordinate the signal is smoothed with a Savitzky–Golay (SG) filter with window size of five and polynomial order of two to reduce jitter from the Kinect tracking while maintaining the shape of the signal (16). The sequence is then linearly resampled with piecewise linear interpolation to a fixed length of 64 frames, without loss of flexibility gestures or fast taps, to ensure that the model input is not exceeded.

The final cohort retains 2,575 sequences after the following deletions: 12 files carrying the ambiguous correctness label of three were removed, and 2 sequences shorter than 16 frames were discarded as incomplete. No file was rejected for tracking failure. The progression therefore reads 2,589 raw files − 12 ambiguous − 2 short = 2,575 retained sequences across 29 distinct subjects, of which 2,047 are labelled correct and 528 are labelled incorrect. The clean tensor used by the sequence model has shape 2,575 × 64 × 25 × 3, while the handcrafted feature matrix used by the classical baselines has shape 2,575 × 58. Random seeds, hyperparameter search spaces and software versions are reported in Section 3.5.

### 3.3 Feature engineering

Three families of handcrafted features were extracted from every cleaned sequence to represent the kinematic structure of the upper-limb, trunk and head movements contained in IntelliRehabDS (17). The first family contains six joint angles computed across time at the elbows, shoulders and knees, together with a neck angle defined by the Neck–SpineShoulder–SpineMid triplet. For every angle, the mean, standard deviation, minimum, maximum and range are recorded. The second family captures temporal dynamics at the wrists, elbows and head through velocity and acceleration magnitudes derived by finite differencing, complemented by a jerk proxy defined as the mean absolute change in acceleration. The third family encodes inter-limb asymmetry through the mean Euclidean distance between matched left and right joints and records the lateral, vertical, and depth ranges of the head relative to the SpineShoulder joint.

### 3.4 Mathematical formulation

The evaluated processing chain can be summarised by the equations below. Equation 1 defines the centred and scaled skeleton sequence, where p_(t,j) is the raw three-dimensional position of joint j at frame t, p_(t, SB) is the SpineBase position at the same frame, and L_torso is the average Euclidean distance between SpineBase and Neck along the sequence.


$$\hat{p}\_(t,j)=(p\_(t,j)-p\_(t,\mathrm{SB}))/L\_\text{torso}$$ (1)

Equation 2 expresses the temporal smoothing through a Savitzky–Golay convolution of length W and polynomial order O, where the kernel coefficients c_k depend only on W and O and are computed once.


p∼_(t,j,c)=Σ_{k=−(W−1)/2}^{(W−1)/2}c_k⋅p∧_((t+k),j,c) (2)

Equation 3 returns a joint angle *θ* at the proximal vertex p2 formed by the bones (p1 − p2) and (p3 − p2), and Equation 4 produces the inter-limb asymmetry index between two homologous joints a and b by averaging frame-wise Euclidean distances over the resampled sequence of length T.


θ_t=arccos[((p1−p2)⋅(p3−p2))/(‖p1−p2‖⋅‖p3−p2‖+ε)] (3)

A_(a,b)=(1/T)⋅Σ_{t=1}^{T}‖p∼_(t,a)−p∼_(t,b)‖ (4)

Equation 5 describes the classification head of the evaluated Spatio-Temporal Skeletal Network (ST-SkelNet) comparator. The temporal feature map H produced by two stacked one-dimensional convolutions is pooled with attention weights α_t computed by a learned scorer, and the resulting context vector is mapped to a sigmoid output that estimates the probability that a movement is incorrect. The parameter w_a is the attention weight vector, w_f and b_f are the final classifier weights, and *σ* is the sigmoid function.


$$\begin{array}{l} \alpha \_t=\text{softmax}\_t(H\_t\cdot w\_a),\\ y=\sigma ((\Sigma \_t\;\alpha \_t\cdot H\_t)\cdot w\_f+b\_f) \end{array}$$ (5)

Equation 6 introduces the skeletal graph-convolution module used by ST-SkelNet. Let A ∈ R^(25 × 25) be the symmetrically normalised adjacency matrix of the Kinect v2 skeleton with self-loops, and X_t ∈ R^(25 × F) the per-joint feature matrix at time t. A graph-convolutional layer with weight W_g and ReLU activation produces the topology-aware feature map Z_t = ReLU(A · X_t · W_g), which is concatenated with the temporal feature map H_t before attention pooling. This module exploits the anatomical connectivity of the skeleton (2, 10).


$$Z\_t=\text{ReLU}(A\cdot X\_t\cdot W\_g),\tilde{H}\_t=[H\_t;Z\_t]$$ (6)

All parameters of the equations described above are listed in Table 2 together with their roles, which clarifies how the algebraic blocks map onto the implementation.

**Table 2:** Notation and parameters used in Equations 1–6.

Symbol	Meaning
p_(t,j)	Raw 3D coordinate of joint j at frame t
p_(t, SB)	Spinebase coordinate at frame t
L_torso	Mean spinebase-to-Neck distance
W, O	Savitzky–Golay window length and polynomial order
c_k	Fixed Savitzky–Golay kernel coefficients
θ_t	Joint angle at frame t
A_(a,b)	Inter-limb asymmetry index for homologous joints a and b
T	Number of resampled frames (set to 64)
H_t	Temporal feature map at step t
α_t	Attention weight at step t
w_a, w_f, b_f	Learnable parameters of attention and classifier
σ	Sigmoid activation
A (Equation 6)	Symmetrically normalised skeleton adjacency matrix (25 × 25)
X_t, Z_t	Per-joint feature matrix and graph-convolution output at time t
W_g	Learnable weights of the skeletal graph-convolution layer

ε denotes a small numerical-stability constant (10^−8^) used in Equation 3.

### 3.5 Models and reproducibility

Five classical baselines are compared with ST-SkelNet as a sequence-based topology-aware comparator. The classical baselines are logistic regression with class-balanced weights, a random forest, a gradient-boosting classifier, a support vector classifier with a radial-basis kernel and class-balanced weights, and a multilayer perceptron (MLP) (18). Each classical learner consumes the 58-dimensional feature vector after fold-contained standardisation. ST-SkelNet operates on a per-frame descriptor of dimension 157, formed by the flattened 75-dimensional joint vector, seven joint angles and a 75-dimensional velocity term. Two stacked one-dimensional convolutions of width five with hidden sizes 48 and 24 encode temporal context. A skeletal graph-convolution layer with hidden size 32 is applied along the joint dimension, and its output is concatenated with the temporal representation before attention pooling. Training uses Adam with a learning rate of 0.001, weight decay 1 × 10^−4^, gradient clipping at norm 1.0, batch size 64 and 15 epochs. Random seeds are fixed at 42 for NumPy, PyTorch and Python. Software versions are Python 3.10, NumPy 1.26, SciPy 1.11, scikit-learn 1.3, imbalanced-learn 0.11, PyTorch 2.1 and Matplotlib 3.8. All candidate search spaces and model-selection criteria were prespecified before outer-fold evaluation; final hyperparameters were selected exclusively within the inner subject-wise cross-validation loops.

To clarify the hyperparameter selection procedure, we expand on the statement above. Hyperparameters were selected by a systematic nested cross-validation procedure that never touched the held-out test fold. Within every outer training fold of the subject-wise five-fold split, an inner three-fold subject-wise cross-validation was run as a grid search; the inner folds were drawn only from the subjects present in the outer training partition, so no test-fold subject ever influenced hyperparameter choice. For the classical baselines the inner grid covered the random-forest tree count {100, 200, 400} and maximum depth {none, 10, 20}, the gradient-boosting learning rate {0.05, 0.1, 0.2} and stage count {100, 200}, the support-vector regularisation C {0.1, 1, 10} with the radial-basis kernel width in {scale, auto}, and the logistic-regression inverse-regularisation C {0.1, 1, 10}. For the multilayer perceptron the inner grid covered the hidden-layer configuration {(64,), (128, 64), (256, 128)}, the activation {ReLU, tanh}, the L2 regularisation *α* {0.0001, 0.001, 0.01}, the learning rate {0.0005, 0.001} and the batch size {32, 64}, with early stopping enabled. To prevent any information leaking from the validation subjects into preprocessing or oversampling, feature scaling and SMOTE were not applied once before the grid search; instead they were placed inside an imbalanced-learn pipeline structured as Scaler → SMOTE → Classifier and refitted within every inner training fold. The outer validation subject therefore never influenced scaling, oversampling, model selection or decision-threshold selection. For ST-SkelNet the inner grid covered the learning rate {0.0005, 0.001, 0.002}, the batch size {32, 64, 128}, the minority-class weight {2.0, 3.0, 4.0} and the graph-convolution hidden size {16, 32, 64}. The configuration maximising the mean inner-fold macro-F1 was then refitted on the full outer training partition and applied once to the untouched outer test fold. The values reported earlier in this section are the modal configurations selected by this procedure across the five outer folds; they were fixed before the outer test folds were scored, which is the sense in which no test-set tuning was performed.

Class imbalance is addressed by two complementary mechanisms. First, the cross-validation splits are subject-wise stratified, meaning that the proportion of incorrect-execution subjects is preserved across folds while no subject appears in both training and test partitions. Second, within each training fold we apply Synthetic Minority Oversampling Technique (SMOTE) to the standardised feature matrix used by the classical baselines, generating synthetic minority samples by interpolating between nearest neighbours of the same class (19). SMOTE is used only for the classical feature-based models and is fitted strictly inside each training fold (as part of the Scaler → SMOTE → Classifier pipeline described above) to prevent leakage. ST-SkelNet does not use SMOTE: because it operates on raw sequences, class imbalance is handled instead by a class-weighted binary cross-entropy loss with a weight of 3.0 on the minority class. Accordingly, the minority-class recall reported for ST-SkelNet in Section 4 is attributable to this class-weighted loss rather than to SMOTE.

### 3.6 Evaluation protocol

Subject identity is a major nuisance variable in skeleton data, so performance was estimated using five-fold StratifiedGroupKFold cross-validation, with every subject assigned to one outer test fold only (20). Within each outer training partition, three-fold subject-wise inner cross-validation selected the configuration that maximised mean macro-F1. Feature scaling and SMOTE were fitted separately within each inner training partition for the classical models; ST-SkelNet used class-weighted binary cross-entropy. The selected configuration was refitted on the complete outer training partition and applied once to the untouched outer test subjects. A fixed decision threshold of 0.50 was used, and no threshold was optimised on pooled outer-test predictions.

For each outer fold, accuracy, precision, incorrect-class recall, specificity, negative predictive value, F1 and ROC-AUC were calculated, and mean ± standard deviation was reported across folds. Every sequence contributed exactly one out-of-fold prediction, allowing pooled confusion matrices, balanced accuracy, macro-F1 and ROC-AUC to be reported for the complete cohort. The incorrect-execution class was treated as positive. The sequence-level bootstrap intervals accompanying pooled ROC-AUC values are descriptive because repeated sequences are clustered within 29 subjects; they are not used to make pairwise superiority claims. Model differences are therefore interpreted through the magnitude and consistency of the reported metrics rather than through unsupported significance statements.

### 3.7 Algorithm description

Algorithm 1 summarises the entire procedure, from raw file ingestion to model evaluation. The algorithm is intentionally written in plain pseudocode so that practitioners outside the machine learning community can audit and reproduce the steps.

Algorithm 1: **Leakage-controlled nested subject-wise evaluation**

Input: skeleton sequences S, labels y, subject identifiers g, outer folds K = 5, inner folds J = 3
Output: out-of-fold predictions and performance estimates
1. Parse the raw skeleton files and remove ambiguous or incomplete sequences.
2. Root-centre and torso-scale each sequence, apply temporal smoothing, resample to 64 frames and extract the predefined kinematic features.
3. Construct five subject-disjoint outer folds using StratifiedGroupKFold.
4. For each outer fold:
a. Reserve the outer test subjects without using them in preprocessing, tuning or threshold selection.
b. Construct three subject-disjoint inner folds from the outer training subjects.
c. For every classical-model configuration, fit Scaler → SMOTE → Classifier only on the current inner-training partition and evaluate it on the inner-validation subjects.
d. For every ST-SkelNet configuration, train with class-weighted binary cross-entropy on the inner-training subjects and evaluate it on the inner-validation subjects.
e. Select the configuration with the highest mean inner-fold macro-F1.
f. Refit the selected configuration on the complete outer-training partition.
g. Predict labels and probabilities once for the untouched outer-test subjects and store sample ID, subject ID, true label, predicted label and predicted probability.
5. Pool the out-of-fold predictions from all five outer folds.
6. Calculate fold-averaged metrics, pooled confusion matrices, balanced accuracy, macro-F1, sensitivity, specificity and ROC-AUC.
7. Report model differences descriptively and avoid clinical or architectural superiority claims unsupported by the benchmark

## 4 Results and analysis

### 4.1 Cohort and class composition

**Figure 1:**
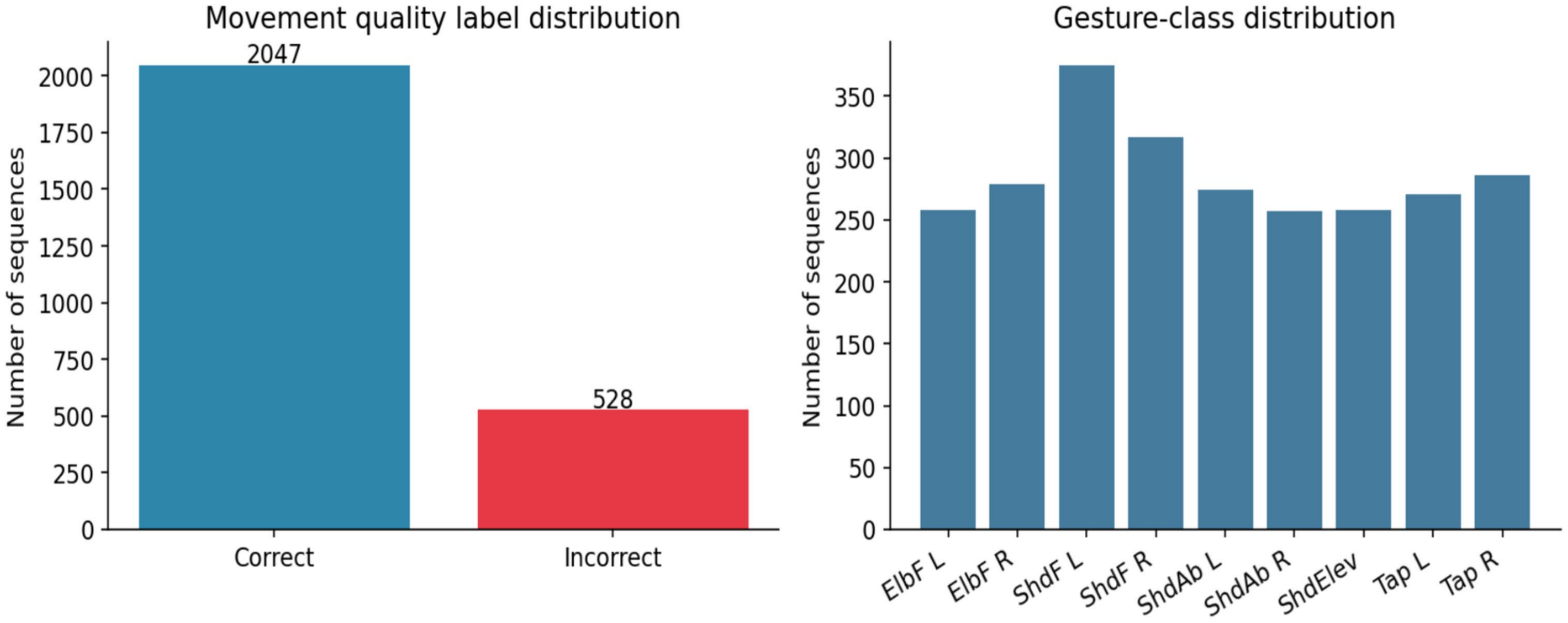
Distribution of the binary correctness label and of the nine gesture classes after pre-processing.

Figure 1 summarises the composition of the clean cohort. The left panel shows that the binary correctness label is imbalanced, with 2,047 sequences labelled correct and 528 labelled incorrect, a ratio close to 4 to 1. The right panel shows that the gesture vocabulary is approximately balanced, with the most frequent class being shoulder flexion of the left arm at 376 sequences and the least frequent being shoulder abduction of the right arm at 257 sequences. Twenty-nine unique subjects are present, distributed between 14 healthy controls and 15 patients according to the original metadata.

### 4.2 Methodology flowchart

**Figure 2:**
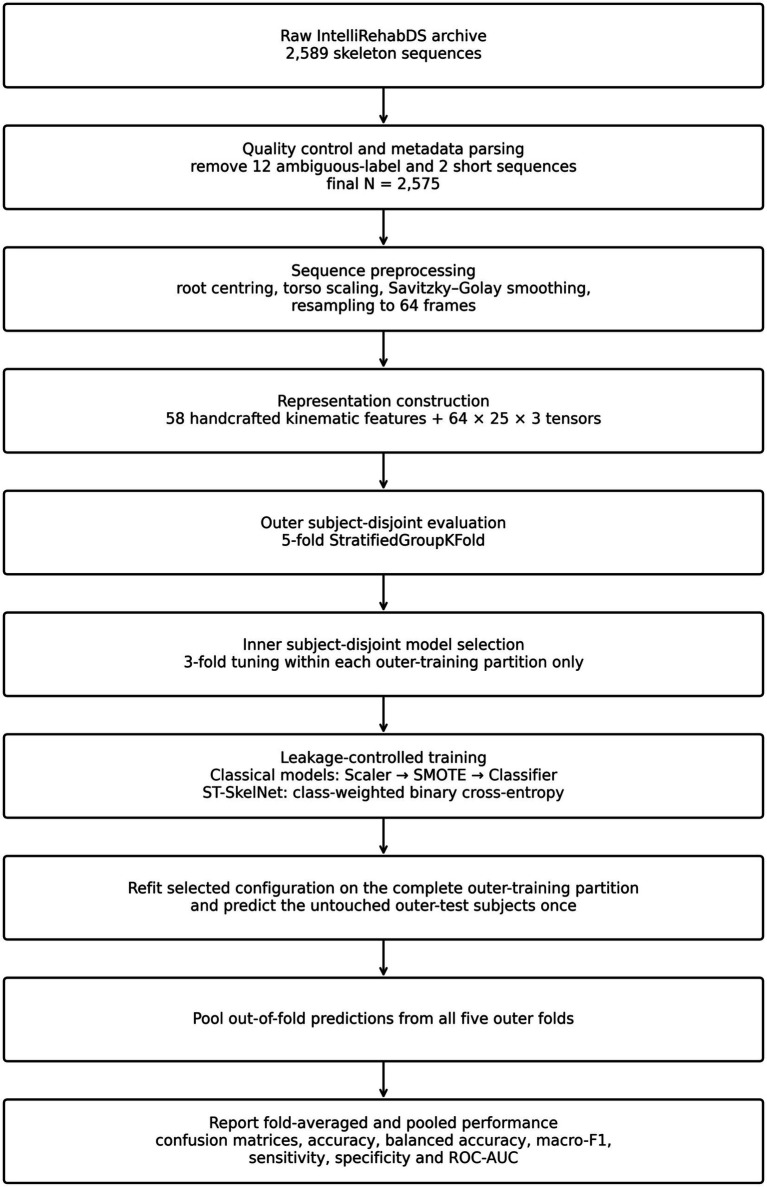
Leakage-controlled nested subject-wise evaluation workflow. Hyperparameters are selected only within the outer-training subjects, and each outer-test sequence contributes one pooled out-of-fold prediction.

Figure 2 summarises the complete leakage-controlled workflow. It distinguishes the outer subject-disjoint evaluation loop from the inner subject-wise model-selection loop and makes explicit that scaling and SMOTE are fitted only for the classical models within training partitions, whereas ST-SkelNet uses class-weighted loss. Each outer-test subject is evaluated once, after which the out-of-fold predictions are pooled for diagnostic reporting. The implementation uses Python 3.10, NumPy 1.26, SciPy 1.11, scikit-learn 1.3, imbalanced-learn 0.11, PyTorch 2.1 and Matplotlib 3.8.

### 4.3 Feature importance

**Figure 3:**
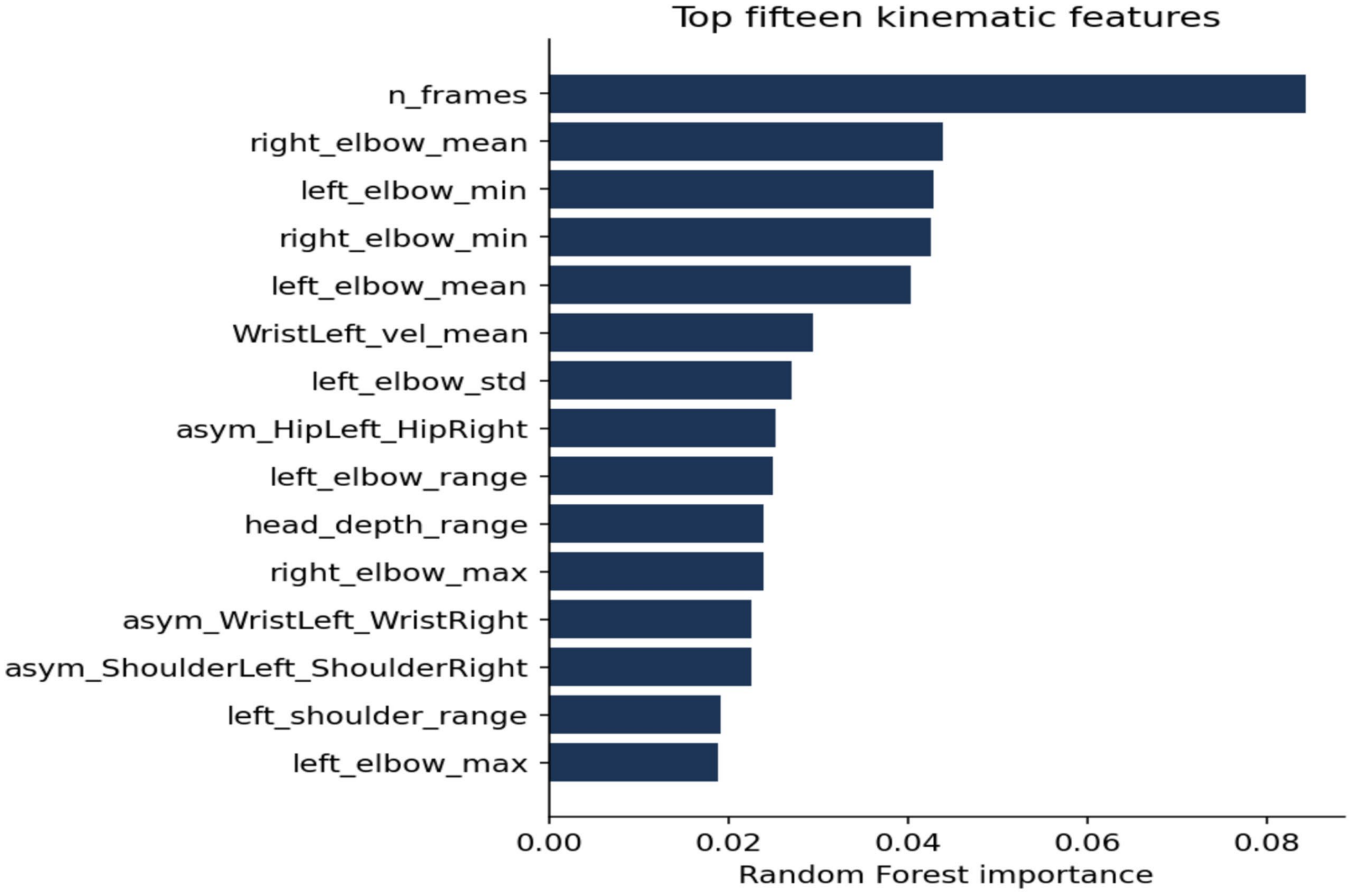
Top 15 features ranked by random-forest impurity-based importance, fitted on the training portion of fold 1 (N_train = 2,060 sequences before SMOTE; 1,638 correct and 422 incorrect, balanced to 3,276 sequences after within-fold SMOTE). Feature values are reported as the mean decrease in Gini impurity averaged across the forest of 200 trees.

Figure 3 displays the 15 most important features under the random forest model, fitted on the training partition of the first outer fold after standardisation. The single most influential feature is the number of frames of the original sequence, which captures the fact that incorrect executions tend to deviate from the prescribed temporal pattern (21). The next most influential features are joint angles at the elbows, in particular the right elbow mean, the left elbow minimum and the right elbow minimum, which is consistent with the fact that the gesture catalogue is dominated by upper-limb tasks. Two asymmetry features, the asymmetry of the wrists and the asymmetry of the shoulders, also appear in the top 15, indicating that bilateral-asymmetry variables were associated with correctness labels within this IntelliRehabDS adult/adolescent cohort. Whether similar variables are informative in infants requires independent paediatric data and cannot be inferred from the present analysis. The depth-axis range of motion of the head also enters the top features, capturing the fact that incorrect executions involve uncontrolled head displacements toward or away from the sensor.

### 4.4 Head range-of-motion patterns

**Figure 4:**
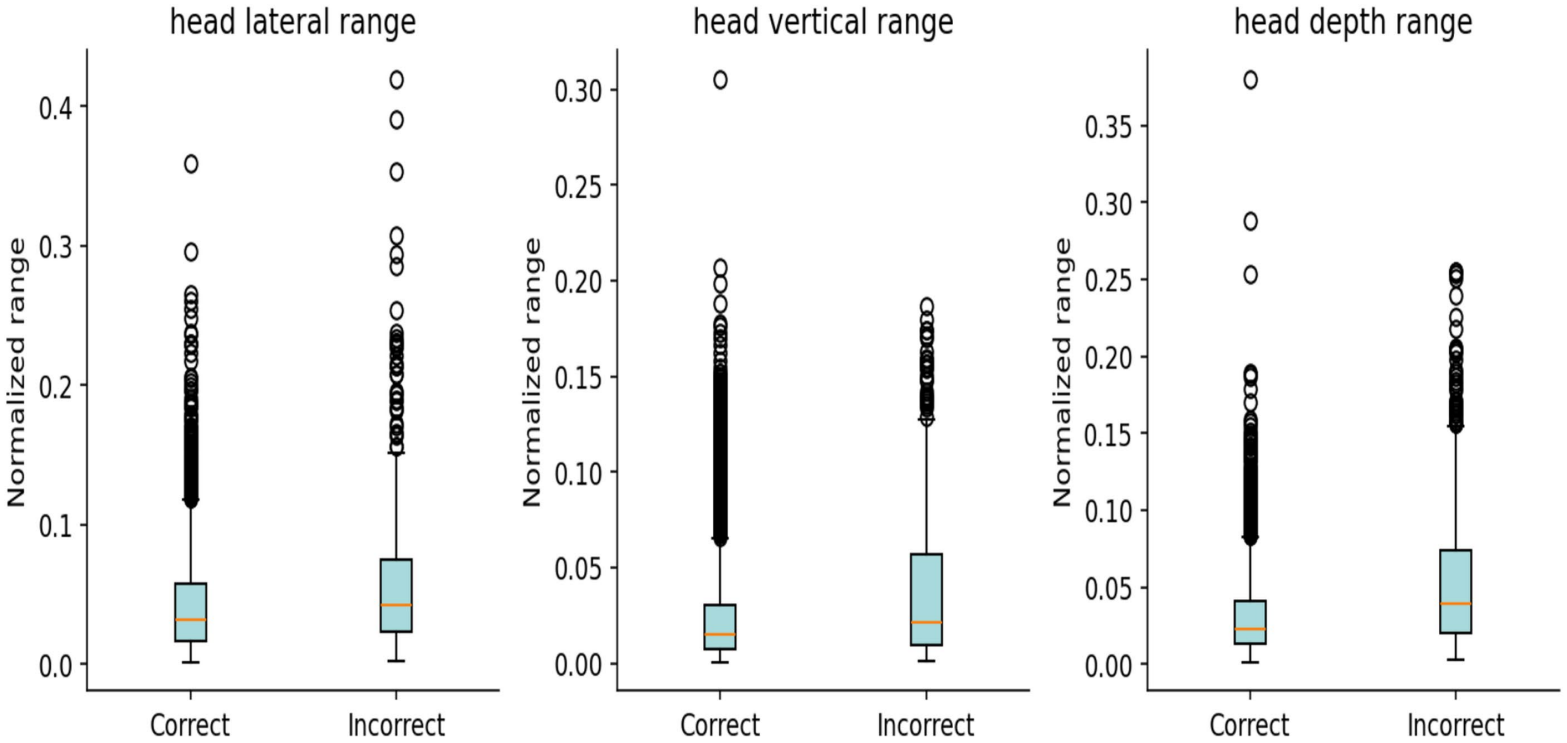
Distribution of lateral, vertical and depth head range of motion for correct (*n* = 2,047) and incorrect (*n* = 528) executions in the IntelliRehabDS adult/adolescent cohort. Boxes show the interquartile range, centre lines show the median, whiskers extend to 1.5 × IQR and individual outliers are plotted.

Figure 4 compares the lateral, vertical and depth ranges of head motion for correct and incorrect executions. Across all three axes, incorrect executions show higher median values, wider interquartile ranges and a larger upper tail than correct executions, with the greatest visual separation on the depth axis. These distributions are presented as an exploratory description of the IntelliRehabDS adult/adolescent cohort rather than as a clinical inference. Because multiple sequences were recorded from each of only 29 subjects, sequence-level *p*-values are not used to claim population-level effects. Whether comparable head-motion patterns are informative in infants requires independently collected paediatric data and cannot be inferred from the present analysis.

### 4.5 Classification performance

Table 3 reports the cross-validated metrics of every model. Among the classical baselines, the multilayer perceptron achieves the highest accuracy at 0.869, followed by the support vector classifier at 0.855 and the gradient-boosting model at 0.851. The random forest reaches 0.837 in accuracy and the highest area under the curve among all models at 0.861. Logistic regression, the simplest baseline, still attains 0.818 in accuracy. The sequence-based topology-aware comparator, ST-SkelNet, reaches 0.846 in accuracy and 0.855 in area under the curve, with a precision of 0.634 and a recall (sensitivity) of 0.612. This recall reflects the class-weighted binary cross-entropy loss used for ST-SkelNet rather than SMOTE, which is applied only to the classical feature-based models. Standard deviations across folds are reported, showing that variability is comparable to that observed by other works using subject-wise evaluation (4).

**Table 3:** Five-fold subject-wise cross-validation results on IntelliRehabDS (*n*, 2,575 sequences from 29 subjects).

Model	Accuracy	Precision	Recall	Specificity	NPV	F1	AUC
Logistic regression	0.818 ± 0.045	0.516	0.612	0.871	0.890	0.560	0.776
Random forest	0.837 ± 0.058	0.682	0.398	0.951	0.851	0.502	0.861
Gradient boosting	0.851 ± 0.052	0.654	0.511	0.939	0.876	0.574	0.823
SVM (RBF)	0.855 ± 0.053	0.592	0.629	0.913	0.901	0.610	0.846
MLP	0.869 ± 0.058	0.646	0.587	0.942	0.895	0.615	0.824
ST-SkelNet	0.846 ± 0.040	0.634	0.612	0.918	0.898	0.623	0.855

Values are presented as mean ± standard deviation across five subject-wise outer folds where available. Recall represents sensitivity for the minority incorrect-execution class. For the classical feature-based models, scaling and SMOTE were fitted exclusively within the relevant training partitions. ST-SkelNet did not use SMOTE; class imbalance was addressed with class-weighted binary cross-entropy. NPV, negative predictive value; specificity, true-negative rate for the correct-execution class; false-negative rate, 1 − recall.

The results do not support a claim that ST-SkelNet is superior to the strongest classical baselines. The MLP attains the highest accuracy (0.869), the random forest the highest ROC-AUC (0.861), and the SVM the highest incorrect-class recall (0.629), whereas ST-SkelNet reaches 0.846 accuracy, 0.855 ROC-AUC and 0.612 recall. ST-SkelNet is therefore interpreted as a competitive topology-aware sequence comparator rather than as a performance advance. The contribution of the manuscript is the leakage-controlled benchmark and the transparent comparison of model trade-offs.

**Figure 5:**
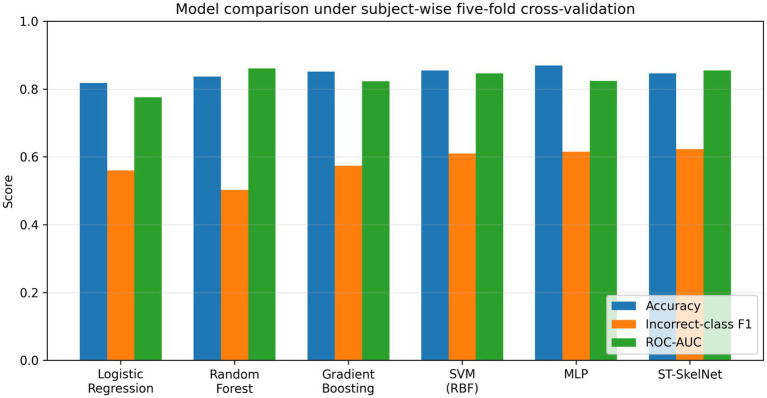
Comparative accuracy, incorrect-class F1 and ROC-AUC of the evaluated models on the IntelliRehabDS adult/adolescent cohort (*n* = 2,575 sequences) under subject-wise five-fold cross-validation.

Figure 5 visualises accuracy, incorrect-class F1 and ROC-AUC for the six evaluated models. The models occupy a relatively narrow accuracy range of 0.818–0.869 and an ROC-AUC range of 0.776–0.861, but they differ substantially in minority-class detection. The false-negative rate ranges from 0.371 for the SVM to 0.602 for the random forest. Consequently, model selection should consider balanced accuracy, macro-F1, recall and specificity rather than headline accuracy alone; these pooled imbalance-aware measures are reported in Table 4.

**Table 4:** Imbalance-aware pooled out-of-fold metrics for all evaluated models (positive class, incorrect execution).

Model	Balanced accuracy	Macro-F1	Incorrect-class recall	Specificity	ROC-AUC
Logistic regression	0.741	0.732	0.612	0.871	0.776
Random forest	0.675	0.703	0.398	0.952	0.861
Gradient boosting	0.725	0.747	0.511	0.939	0.823
SVM (RBF)	0.771	0.774	0.629	0.913	0.846
MLP	0.764	0.784	0.587	0.942	0.824
ST-SkelNet	0.765	0.772	0.612	0.918	0.855

Balanced accuracy, (incorrect-class recall + specificity)/2. Macro-F1 is the unweighted mean of the F1 scores for correct and incorrect executions. Incorrect-class recall is sensitivity to incorrect executions. Accuracy alone is not used as the primary model-selection measure under the approximately 4:1 class imbalance.

### 4.6 Full confusion-matrix analysis

**Figure 6:**
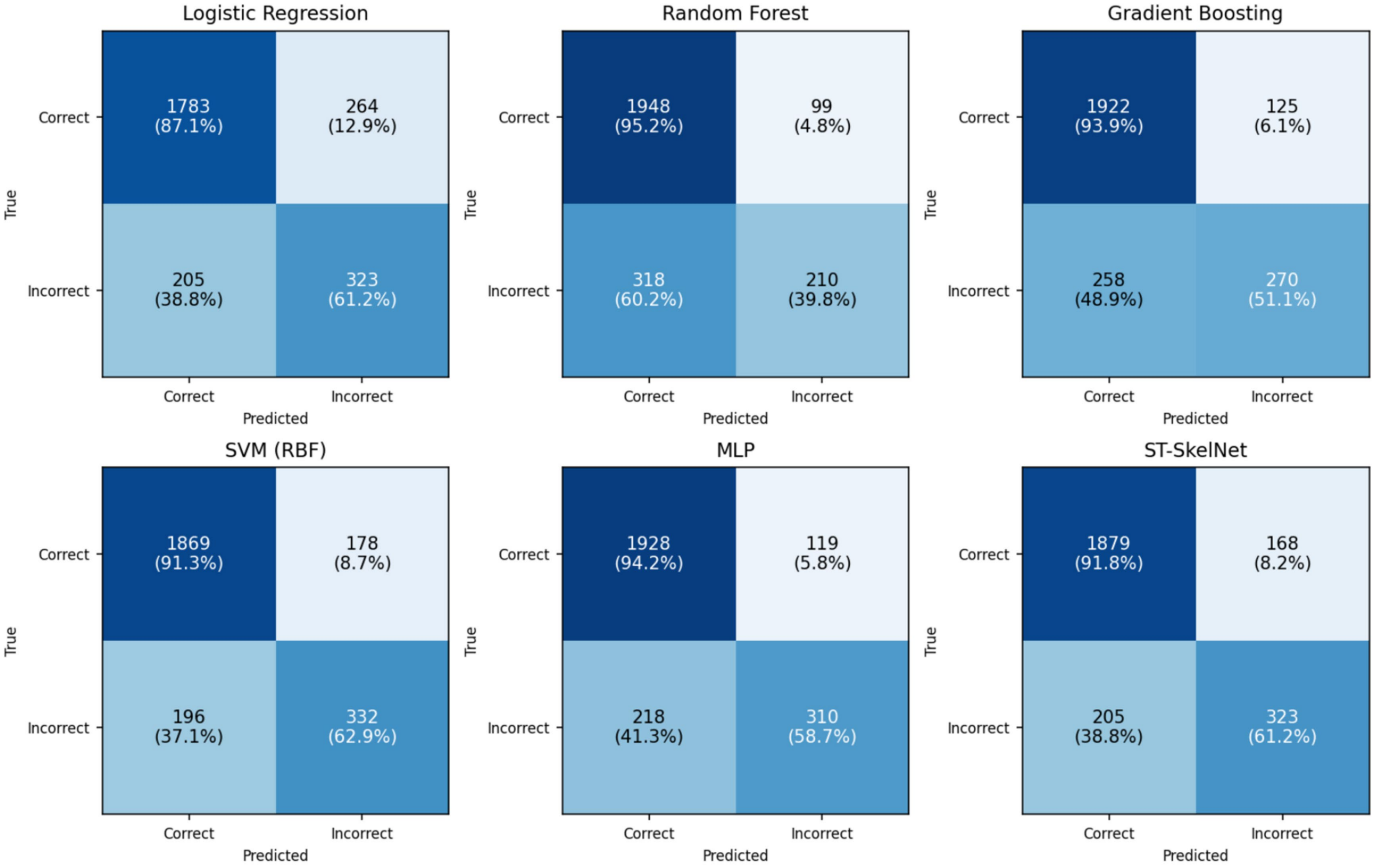
Pooled out-of-fold confusion matrices for Logistic Regression, Random Forest, Gradient Boosting, SVM, MLP and ST-SkelNet under five-fold subject-wise cross-validation. Each cell reports the absolute count and row-normalised percentage. The incorrect-execution class is treated as positive.

Table 5 and Figure 6 report pooled out-of-fold confusion matrices for all six evaluated models. The random forest is the most conservative classifier, identifying 1,948 of 2,047 correct executions but only 210 of 528 incorrect executions, corresponding to specificity 0.952 and incorrect-class recall 0.398. The SVM provides the highest incorrect-class recall (0.629), while the MLP combines high specificity (0.942) with the highest pooled accuracy. ST-SkelNet attains recall 0.612 and specificity 0.918. These results demonstrate that the principal model trade-off is not simply overall accuracy, but the balance between detecting incorrect executions and avoiding false alarms.

**Table 5:** Pooled out-of-fold confusion matrices for all evaluated models (*n*, 2,575 sequences; positive class, incorrect execution).

Model	Correct → Correct (TN)	Correct → Incorrect (FP)	Incorrect → Correct (FN)	Incorrect → Incorrect (TP)	Sens.	Spec.
Logistic regression	1783	264	205	323	0.612	0.871
Random forest	1948	99	318	210	0.398	0.952
GRADIENT BOOSTING	1922	125	258	270	0.511	0.939
SVM (RBF)	1869	178	196	332	0.629	0.913
MLP	1928	119	218	310	0.587	0.942
ST-SkelNet	1879	168	205	323	0.612	0.918

TN, FP, FN and TP denote true-negative, false-positive, false-negative and true-positive counts. Sens., sensitivity for incorrect executions; Spec., specificity for correct executions.

### 4.7 ROC-AUC and imbalance-aware performance

Table 5 reports the pooled out-of-fold cells obtained after every sequence was predicted once in its outer test fold. These pooled metrics are not identical to the unweighted fold means in Table 3. Table 3 gives equal weight to each of the five folds, whereas pooled calculations give equal weight to each sequence. For example, ST-SkelNet has a pooled accuracy of 2,202/2,575 = 0.855, compared with an unweighted mean fold accuracy of 0.846. Both summaries are retained: the fold mean describes variability across subject groups, while the pooled matrix describes overall cohort-level classification behaviour.

**Figure 7:**
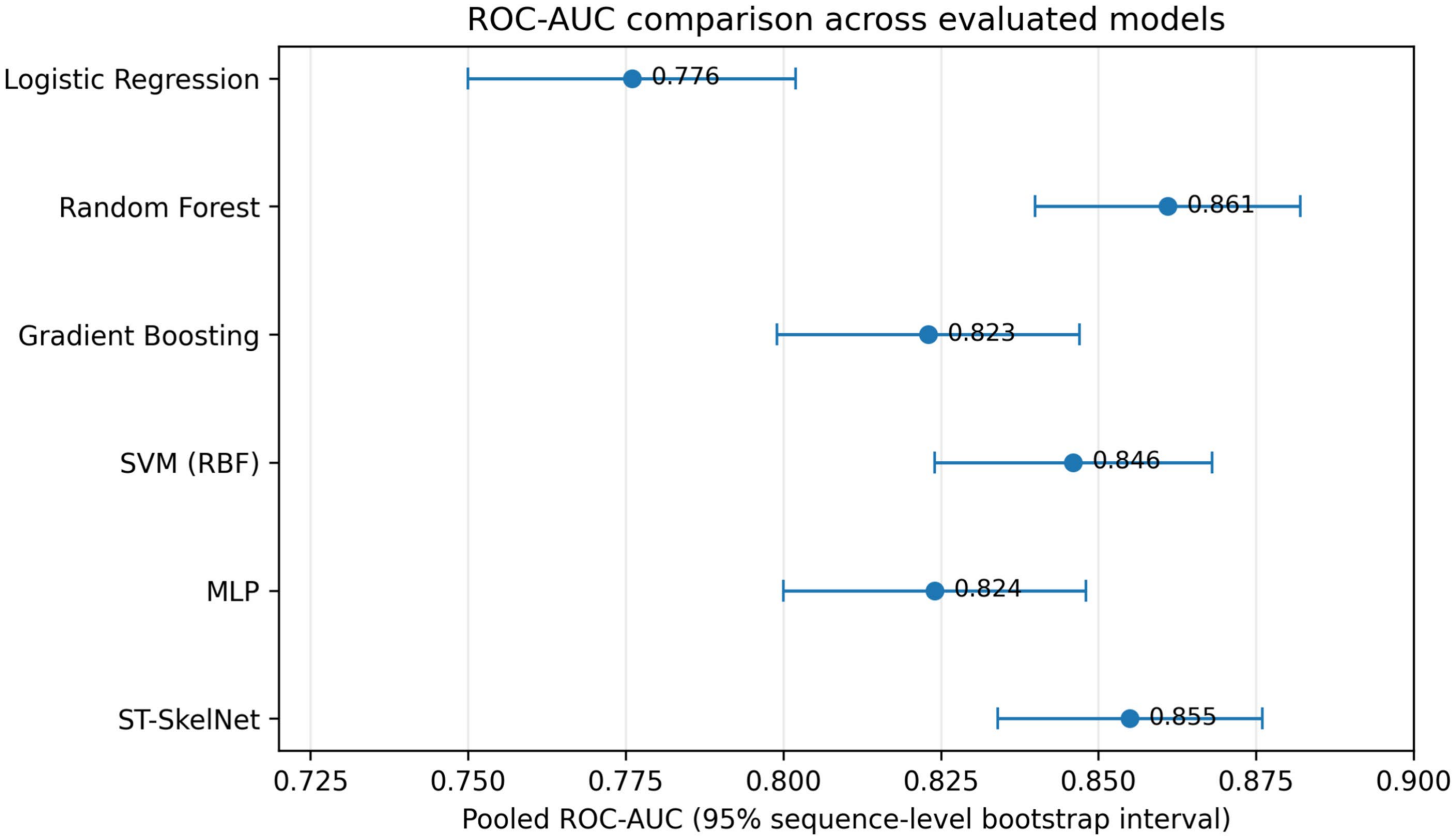
Pooled ROC-AUC estimates and sequence-level bootstrap 95% intervals for the six evaluated models. The incorrect-execution class is treated as positive. The intervals summarise pooled discrimination uncertainty and are not interpreted as pairwise significance tests.

ROC-AUC values are reported in Table 6 and Figure 7 with the incorrect-execution class treated as positive. The random forest achieves the highest pooled ROC-AUC (0.861), followed by ST-SkelNet (0.855) and the SVM (0.846). Because this is a binary task, a single positive-class ROC-AUC is sufficient; duplicate class-specific and macro columns would contain the same value. The accompanying bootstrap intervals were calculated at sequence level and are presented descriptively. They do not establish equivalence or superiority between models because observations are clustered within subjects and no paired subject-level AUC comparison is available.

**Table 6:** Pooled out-of-fold ROC-AUC values for all evaluated models on IntelliRehabDS (positive class, incorrect execution).

Model	ROC-AUC	95% CI
Logistic regression	0.776	0.750–0.802
Random forest	0.861	0.840–0.882
Gradient boosting	0.823	0.799–0.847
SVM (RBF)	0.846	0.824–0.868
MLP	0.824	0.800–0.848
ST-SkelNet	0.855	0.834–0.876

Confidence intervals are sequence-level bootstrap intervals based on 2,000 resamples and are reported descriptively rather than as paired model-comparison tests.

Table 4 complements ROC-AUC with metrics that are sensitive to the approximately 4:1 class imbalance. The SVM and ST-SkelNet provide the highest balanced accuracy (0.771 and 0.765), the MLP the highest macro-F1 (0.784), the SVM the highest incorrect-class recall (0.629), and the random forest the highest specificity and ROC-AUC but the lowest recall. No single model therefore dominates all evaluation criteria. The appropriate model depends on whether the primary objective is sensitivity to incorrect executions, specificity or overall discrimination.

## 5 Discussion

The results demonstrate that the quality of rehabilitation movement can be classified from 3D skeleton data of the adult/adolescent IntelliRehabDS cohort, with the best model achieving an accuracy of 0.869 in a strictly subject-disjoint evaluation. This finding is only to be understood as an indication of the benchmark task. It does not imply any evidence concerning infants with congenital muscular torticollis. The discussion below clearly distinguishes conclusions drawn from the adult/adolescent experiments from those that would depend on paediatric data.

First, the most important features from the random forest analysis are predominantly joint angles and the bilateral-asymmetry indices. These variables are attractive for interpretability because they can be inspected directly in the feature space rather than through a black-box representation, and within this adult/adolescent cohort they were associated with the correctness labels. We cannot assume the same variables would be diagnostic in any clinical group; their informativeness in infants needs to be assessed independently on paediatric data and cannot be extrapolated from the present analysis.

Second, descriptive separation between correct and incorrect executions is displayed in the lateral, vertical and depth head-motion ranges in Figure 4. The shift on the depth axis is most apparent and the scatter of incorrect executions is the largest. These distributions do not represent population-level causal or clinical effects, because the data comprise repeated sequences from only 29 participants. They show only that “head-motion variables” provide information regarding correctness labels in IntelliRehabDS.

Thirdly, there is no dominant model. The competitive discrimination in ST-SkelNet is not reflected in increased accuracy when compared with MLP (0.846 vs. 0.869) or ROC-AUC when compared with random forest (0.855 vs. 0.861), so it is reported not as a superior architecture, but as a topology-aware sequence comparator. This pattern is consistent with recent benchmarking studies (13), which report only limited gains from deep rehabilitation models in small-subject settings. In the present study, all quantitative conclusions are based on a single protocol, the same data, the same folds, and the same data preprocessing, tuning criteria and metrics. The results demonstrate that classical learners that are easy to audit remain competitive with the sequence model, and that a more interpretable model is a reasonable default when its performance is comparable.

Fourth, comparisons with published benchmarks should not be taken at face value, because those studies are based on different datasets, tasks and evaluation measures. For example, the error-classification result reported for the PhysioFormer study on KERAAL and that of the best model in the present study (approximately 0.74 versus 0.869 accuracy) differ in context rather than in a like-for-like comparison. Other studies in Table 1 use different measures of continuous quality (mean absolute deviation or rank correlation) and their headline score cannot be directly compared to the binary accuracy and ROC-AUC in the present study. It is not the higher cross-dataset score that is the methodological contribution of this study, but rather a clear correctness benchmark (IntelliRehabDS) which is evaluated in a strictly subject-disjoint manner.

Fifth, the confusion matrices show that some of the models are more sensitive than specific. This is especially true of the random forest, which achieves the highest specificity of 0.952 and the lowest incorrect-class recall of 0.398. Overall accuracy can mask poor minority-class detection, as demonstrated by this operating pattern, which is maintained throughout the fold for the classical models and class-weighted loss for ST-SkelNet. Therefore, in addition to ROC-AUC, balanced accuracy, macro-F1, recall and specificity should be reported. No claims are made regarding caregiver-facing tools, screening utility or therapy planning, because the present adult/adolescent experiments do not support them.

### 5.1 Future research: a possible paediatric validation direction

The present benchmark is conducted entirely on adult and adolescent data, and none of its results constitute evidence about infants. As a future direction only, and not as validation evidence or as a contribution of the present paper, we outline a concise three-stage framework through which the leakage-controlled protocol established here could subsequently be transferred to a paediatric setting. Stage 1 (data collection): assemble an infant-specific dataset using a child-friendly capture protocol, with execution quality independently annotated by paediatric physiotherapists and reported inter-rater agreement. Stage 2 (paediatric model development): re-apply the present subject-disjoint, leakage-controlled nested cross-validation on that infant cohort, refitting all preprocessing, imbalance handling and model selection strictly within paediatric training partitions so that no infant subject appears in both training and test folds. Stage 3 (prospective external clinical validation): evaluate the resulting models prospectively on an independent paediatric cohort against clinician-defined outcomes before any clinical or screening use is considered. Each stage requires its own dataset, ethics approval and clinical oversight; any conclusion about congenital muscular torticollis would depend entirely on this future work and on prospective clinical validation, not on anything reported in the present study. This staged framework is therefore explicitly future research and does not constitute a machine-learning contribution of the present paper, whose scope remains the adult/adolescent methodological benchmark.

### 5.2 Limitations

Several limitations remain. First, IntelliRehabDS contains only 29 distinct subjects, so the precision and generalisability of subject-level performance estimates are limited despite the use of subject-disjoint cross-validation. Independent replication on a larger and more diverse cohort is required. Second, the cohort consists of adults and adolescents rather than paediatric participants; the findings therefore cannot be extrapolated to infant biomechanics or congenital muscular torticollis. Third, the gesture catalogue does not contain infant-specific cervical mobilisation tasks, and execution quality is represented by a binary label rather than a graded clinical score (5). Fourth, Kinect v2 skeleton tracking may degrade under occlusion and does not reproduce infant anatomical proportions. Fifth, the labels represent immediate execution correctness rather than long-term functional or clinical outcomes. Finally, the study evaluates one public dataset and does not include an external validation cohort, so the reported benchmark should be interpreted as internal methodological evidence only.

## 6 Conclusion

This study provides a reproducible, leakage controlled gold standard for the rehabilitation movement-quality classification of skeleton-based motion using the IntelliRehabDS adult/adolescent cohort. The evaluation is realized through a subject-disjoint nested cross validation framework, additionally fold-contained preprocessing and imbalance handling are used, and a direct comparison of five classical feature-based models is performed with the topology-aware ST-SkelNet sequence model. Based on the obtained results, it is observed that the performance of the models is not consistent across all the evaluation metrics, the multilayer perceptron model gives the highest accuracy, the random forest model has the highest ROC-AUC, and ST-SkelNet is also performing well but is not the best of all the models. The results highlight the need for sensitivity, specificity, balanced accuracy, macro-F1, and ROC-AUC as well as accuracy.

The study contribution is not architectural nor clinical, but methodological. It offers a well-defined environment for assessing rehabilitation movement classifiers and mitigates leakage of subjects, model selection bias and class imbalance. The conclusions are limited to those movements in IntelliRehabDS of adults and adolescents. Access to the congenital muscular torticollis database or any other paediatric dataset would involve collection of an age-specific dataset by another application and would be prospective and clinically validated by clinicians, and would therefore not be available for use at this time.

## Data Availability

The original contributions presented in the study are included in the article/supplementary material, further inquiries can be directed to the corresponding author.
